# Multi-dichotomous sequencing method MDSM for ordering the importance of variants’ properties in multi-criteria decision-making

**DOI:** 10.1016/j.mex.2023.102538

**Published:** 2023-12-26

**Authors:** Hubert Anysz, Bartosz Grucza

**Affiliations:** a Warsaw University of Technolgy, Faculty of Civil Engineering, Al. Armii Ludowej 16, Warsaw 00-637, Poland; b Warsaw School of Economics, Department of Infrastructure and Mobility Studies, Al. Niepodległości 162, Warsaw 02-554, Poland

**Keywords:** Multi-Dichotomous Sequencing Method MDSM, Sequencing, Ordering, Algorithms, Multi-criteria decision-making, MCDM, Preference ordering

## Abstract

There are plenty of Multi-Criteria Decision-Making (MCDM) methods that help to choose the most suitable solution assessed by several criteria (e.g. Saaty 1990; Simos 1990; Pamučar et al. 2018). They are applied in cases where several scales of different units describe the variants or the variants' properties are represented by linguistic, non-numbered terms. The inherent part of the MCDM algorithms is calculating the weights of the variants' properties, necessary for ordering the variants. If - in a certain problem - there are several properties to consider, sequencing their importance becomes a problem itself. The innovative method of sequencing is proposed in the article based on dichotomous splitting of the properties' importance. If made several times, it leads to the coherent - internally and with the decision-maker's intention - order of the properties' importance. Then the weights of the properties can be calculated with the use of different MCDM methods. The description of the method can be shortened as follows:

• Divide the full set of features into two dichotomous subsets of lower and higher importance
• Continue dichotomous divisions until there are only the subsets containing one element or subsets containing elements of equal importance

## Graphical abstract

**Figure:**
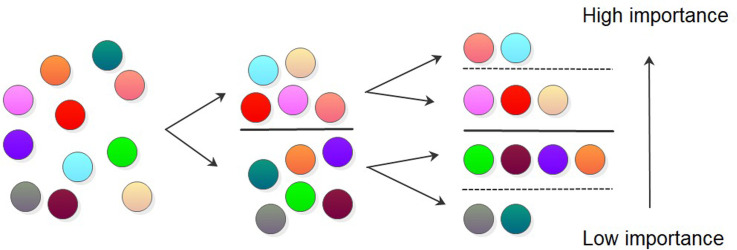

Specifications Table

**Table:** 

Subject area:	Mathematics and Statistics
More specific subject area:	Ordering classes of linguistic variables
Name of your method:	Multi-Dichotomous Sequencing Method MDSM
Name and reference of original method:	T. L. Saaty. How to make a decision: The analytic hierarchy process. „European Journal of Operational Research”. Volume 48. 1, s. 9–26, 1990 J. Simos, Evaluer l'impact sur l'environnement, une approche originale par l'analyse multicritère et la négociation. 1990, 261 p., ISBN 2–880–74,185–8 Pamučar D, Stević Ž, Sremac S. A New Model for Determining Weight Coefficients of Criteria in MCDM Models: Full Consistency Method (FUCOM). Symmetry. 2018; 10(9):393. 10.3390/sym10090393
Resource availability:	Not applicable

## Method details

Ordering the features based on their importance is an easy task if there is a limited number of features. Three or four, even five features can be ordered easily due to their importance for the decision-maker. Let's label the features of any variant as A, B, C, then ordering them according to their importance can be written with the following inequation:

(1) A>C>B

Where the sign > informs about the higher importance of the feature placed on its left side, than of the feature placed on its right side. However, if there are several features (e.g. 15), setting the sequence of the features’ importance is difficult. To make the process of ordering the importance reliable Analytic Hierarchy Process (AHP) was introduced by Saaty [1]. The order of importance is found there through pairwise comparisons of all possible pairs of features. If done for the three features A, B, and C, in Eq. (1) can be expressed in the form of the following three inequations:

(2) A>C

(3) A>B

(4) C>B

Nevertheless, if there are several features, a decision-maker can compare features’ importance inconsistently, for example as

(5) A>C

(6) B>A

(7) C>B

In Eqs. (5)–(7) lead to false in Eq. (8).

(8) B>A>C>B

AHP allows for partial inconsistency in pairwise comparisons of features’ importance. It is a kind of proof, that the ordering of several features is not trivial. If the level of inconsistent assessments is too high, the AHP algorithm stops calculations. Multi-criteria decision-making (MCDM) method, introduced by Simos [2], assumes – as the first step of features’ weights calculations – simply ordering features omitting the difficulty of this process. Also, FUCOM method introduced by Pamucar et al. [3] assumes identical the first step i.e. ordering the features according to their importance.

**Fig. 1:**
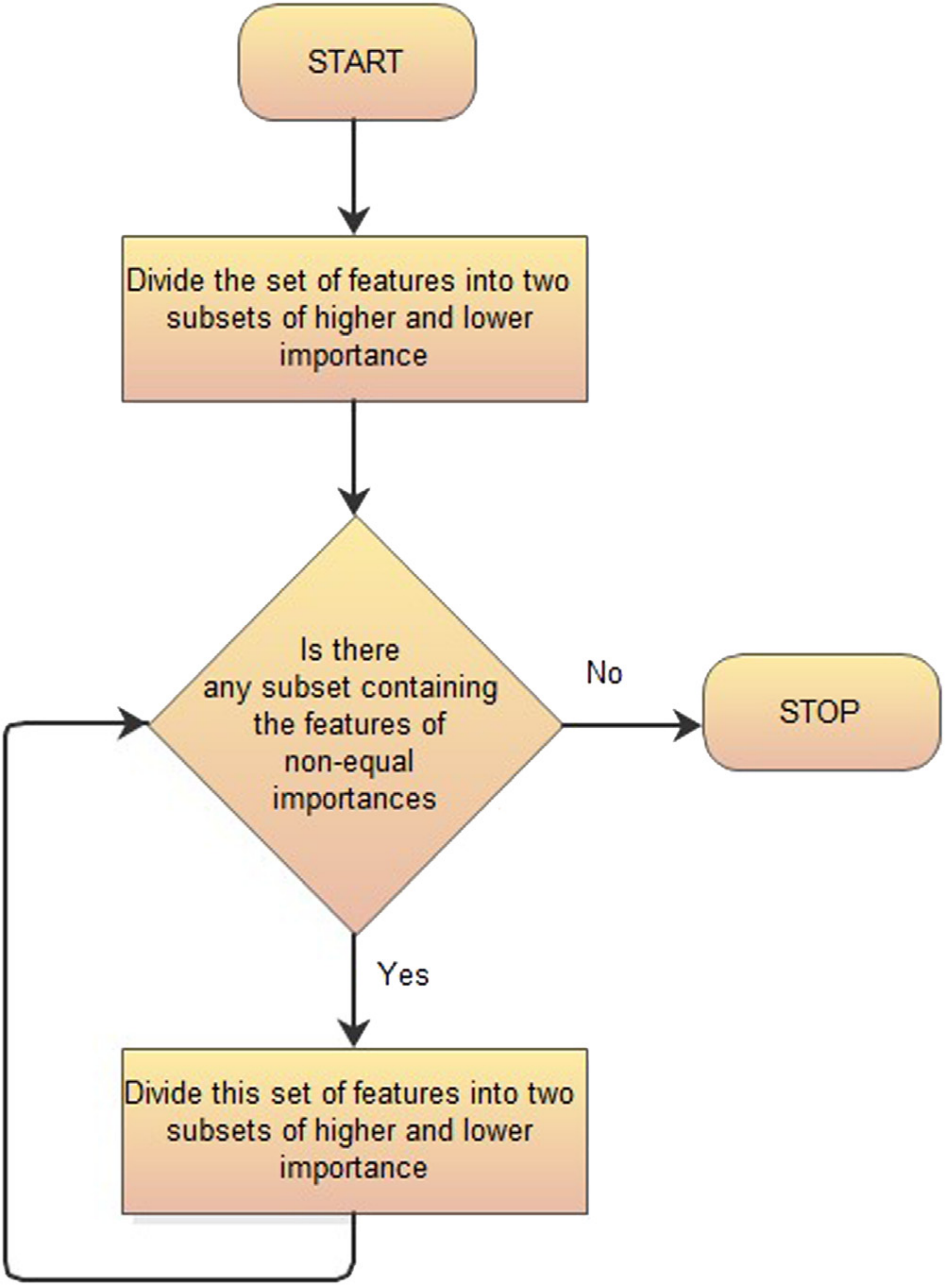
The algorithm of the Multi-Dichotomous Sequencing Method MDSM.

Ordering features’ importance can be executed easily, even for a relatively high number of features with the use of the algorithm presented in Fig. 1.

**Fig. 2:**
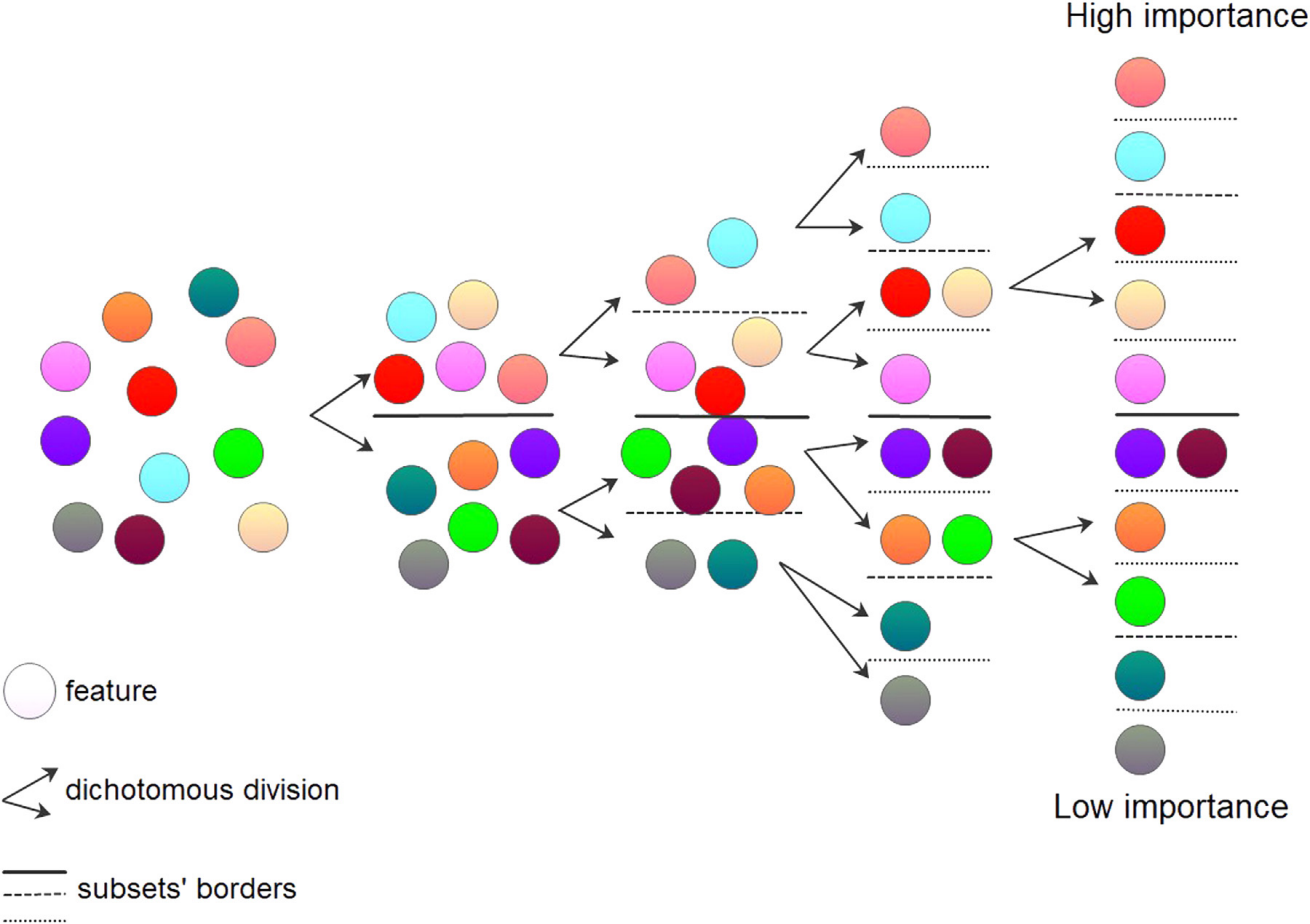
Multi-dichotomous divisions of the full set of features and its subsets. The brown and violet features are of the same importance.

It is much easier for a decision-maker to divide a set of features into two subsets of lower, and higher importance than to make a sequence deciding on the importance of several features. If the dichotomous division is applied several times to all subsets, it creates a consistent ranking of features according to their importance. The scheme of the MDSM applied for the exemplary set of 11 features (represented by coloured circles) is presented in Fig. 2.

Having the features ordered by applying the proposed method (MDSM) the other part of the analysed multi-criteria decision-making method can be executed. In the Simos’ method, the blank cards can be inserted between subsets to increase the distance of importance between analysed features. The next step in AHP is multiple pairwise comparison between all features (expressed in ratios 9:1, 7:1, 5:1, 3:1, 1:1, 1:3, 1:5, 1:7, 1:9). The FUCOM method simply starts with the ordering of the features (criteria). Then, in all these three methods calculation of features’ weights can be continued. By applying MDSM in the Simos’ method and FUCOM the initial order of features’ importance can be achieved. The method is especially helpful for AHP, while this method suffers from inconsistent pairwise comparisons of the considered criteria. Creating the ranking of criteria (importance of features) and then assessing them with the use of the aforementioned ratios provide consistency in criteria assessment and make the use of AHP possible.

## Example of MDSM application

Let's assume that from variety of MCDM tools [4] Simple Additive Weighting (SAW) method – a multi-criteria decision-making method [5,^6^,7] – was chosen to assess the level of sustainability of any building. Then, based on the following SAW formula (9), the level of sustainability could be assigned as a single number to any building.

(9) $$V_{j}=\sum_{i=1}^{n}w_{i}*v_{i}^{\left(j\right)}$$

where:

$V_{j}$ - sustainability level of a building j

n- the total number of criteria applied to the assessment

$w_{i}$ - the weight of i criterion

$v_{i}^{\left(j\right)}$ - the value of assessment of criterion i for a building j (percent from 0 to 100, 100 % means the best)

The weights remains unchanged for all buildings and they should meet the following formula (10).

(10) $$\sum_{i=1}^{n}w_{i}=1$$

Sustainability of a building can be assessed through the following criteria (features, properties of a building) e.g.:

- $CO_{2}$ footprint of construction process (lets mark this property of a building as $p_{1}$) [8]
- $CO_{2}$ yearly footprint of the building ($p_{2}$)
- the share of reused construction materials in the construction process ($p_{3}$)
- the share of highly sustainable, new construction materials ($p_{4}$)
- the level of thermal insulation of walls and a roof ($p_{5}$)
- the yearly energy consumption for heating purposes ($p_{6}$)
- the total yearly energy consumption ($p_{7}$)
- the idle-time power consumption (i.e. during of not operating time, night time) ($p_{8}$)
- the level of natural light usage for room lighting purposes ($p_{9}$)
- the level of utilizing external air temperature to adjusting internal temperature ($p_{10}$)
- the energy produced by the systems installed in a building (e.g. photovoltaic panels) ($p_{11}$)
- the share of rain water used for operating a building ($p_{12}$)
- the size of yearly sewage ($p_{13}$)
- the yearly tonnage of wastes ($p_{14}$)
- the share of green area in the total area of the plot ($p_{15}$)
- the level of engagement in promoting sustainable behaviors among permanent users and visitors of a building ($p_{16}$)
- the number of introduced procedures supporting sustainability ($p_{17}$)

The aforementioned list is not complete. It can be much wider. To apply SAW method, each criterion should has weight. If Simos method is chosen to calculate weights, it is necessary to order these 17 features according to their importance. It is proposed to do so with MDSM i.e. by several dichotomous divisions. Each of the properties should be assigned to one of two subsets: $S_{L}$ of lower importance or $S_{H}$ comprising criteria of higher importance. According to the authors’ preferences the result can be like that: $S_{H}:\;\left\{p_{2},p_{6},p_{8},p_{9},p_{14},p_{16},p_{17}\right\}$ and $S_{L}:\;\left\{p_{1},p_{3},p_{4},p_{5},p_{7},p_{10},p_{11},p_{12},p_{13},p_{15}\right\}.\;$Then it is to check if any of the created subsets contains properties of not equal importance. If yes, the new dichotomous division should be done. Then $S_{H}=S_{HH}\cup S_{HL}$ where (according to the authors) $S_{HH}:\;\left\{p_{2},p_{6},p_{8}\right\}$ and $S_{HL}:\;\left\{p_{9},p_{14},p_{16},p_{17}\right\}$. The subset $S_{H}$ is divided into two dichotomous subsets of higher $S_{HH}$and lower importance $S_{HL}$. Similarly, $S_{L}$ is a source of two its subsets $S_{LH}:\;\left\{p_{1},p_{7},p_{10},p_{11},p_{12},p_{13},p_{15}\right\}$ with the higher importance and $S_{LL}:\;\left\{p_{3},p_{4},p_{5}\right\}$ with the lower importance. Now, there are four sets to check if their elements are of equal importance (within a given set). If not the divisions should be continued up to the moment when each of the subsets is a one-element set or it contains the properties of equal importance. That is the end of MDSM and the ranking of the properties can be presented (see Table 1). For easier following the authors’ dichotomous divisions Table 2 is prepared.

**Table 1:** Dichotomous divisions of the properties.

Features’ importance (high to low)	Not divided yet	Subsets	Subsets	Subsets	Subsets	Ordered Criteria	Features’ importance (high to low)
HIGH	The full set of 17 criteria	$S_{H}$	$S_{HH}$	$S_{HHH}$:		$\left\{p_{6},p_{8}\right\}$	HIGH
				$S_{HHL}$:		$\left\{p_{2}\right\}$	
			$S_{HL}$	$S_{HLH}$	$S_{HLHH}$:	$\left\{p_{14}\right\}$	
					$S_{HLHL}$:	$\left\{p_{9}\right\}$	
				$S_{HLL}$:		$\left\{p_{16},p_{17}\right\}$	
		$S_{L}$	$S_{LH}$	$S_{LHH}$	$S_{LHHH}$*:*	$\left\{p_{7}\right\}$	
					$S_{LHHL}$*:*	$\left\{p_{12},p_{13}\right\}$	
				$S_{LHL}$	$S_{LHLH}$*:*	$\left\{p_{10},p_{11}\right\}$	
					$S_{LHLL}$*:*	$\left\{p_{1}\right\}$	
			$S_{LL}$	$S_{LLH}:$		$\left\{p_{5}\right\}$	
LOW				$S_{LLL}:$		$\left\{p_{3},p_{4}\right\}$	LOW

**Table 2:** Assignment of the properties to the subsets created by dichotomous divisions.

Features’ importance (high to low)	Not divided yet	Subsets	Subsets	Subsets	Ordered Criteria	Features’ importance (high to low)
HIGH	$\left\{\begin{array}{c} p_{1},p_{2},\\ p_{3},p_{4},\\ p_{5},p_{6},\\ p_{7},p_{8},\\ p_{9},p_{10},\\ p_{11},p_{12},\\ p_{13},p_{14},\\ p_{15},p_{16},\\ p_{17}\; \end{array}\right\}$	$\left\{\begin{array}{c} p_{2},p_{6},\\ p_{8},\\ p_{9},p_{14},\\ p_{16},\\ p_{17} \end{array}\right\}$	$\left\{\begin{array}{c} p_{2},p_{6},\\ p_{8} \end{array}\right\}$		$\left\{p_{6},p_{8}\right\}$	HIGH
				:	$\left\{p_{2}\right\}$	
			$\left\{\begin{array}{c} p_{9},p_{14},\\ p_{16},p_{17} \end{array}\right\}$	$\left\{p_{9},p_{14}\right\}$	$\left\{p_{14}\right\}$	
					$\left\{p_{9}\right\}$	
					$\left\{p_{16},p_{17}\right\}$	
		$\left\{\begin{array}{c} p_{1},p_{3},\\ p_{4},p_{5},\\ p_{7},p_{10},\\ p_{11},p_{12},\\ p_{13},p_{15} \end{array}\right\}.$	$\left\{\begin{array}{c} p_{1},p_{7},\\ p_{10},p_{11},\\ p_{12},p_{13} \end{array}\right\}$	$\left\{\begin{array}{c} p_{7},p_{12},\\ p_{13} \end{array}\right\}$	$\left\{p_{7}\right\}$	
					$\left\{p_{12},p_{13}\right\}$	
				$\left\{\begin{array}{c} p_{1},p_{10},\\ p_{11} \end{array}\right\}$	$\left\{p_{10},p_{11}\right\}$	
					$\left\{p_{1}\right\}$	
			$\left\{\begin{array}{c} p_{3},p_{4},\\ p_{5} \end{array}\right\}$		$\left\{p_{5}\right\}$	
LOW					$\left\{p_{3},p_{4}\right\}$	LOW

Having the properties (criteria) ordered from the most important to the properties of the lowest importance, calculation of their weights is possible. The FUCOM method can be applied directly (for details refer to [3]). Simos’ calculations of the weights requires inverting high-low sequence to low high order. They are shortly presented in Table 3.

**Table 3:** Calculation of weights according to Simos' method.

Position in the importance ranking (1 means the lowest)	Cards (properties, criteria)	Quantity of cards	Card numbers	Non-normalized weights	Normalized weights
1	$\left\{p_{3},p_{4}\right\}$	2	1, 2	1,5	0,893
2	$\left\{p_{5}\right\}$	1	3	3	1786
3	$\left\{p_{1}\right\}$	1	4	4	2381
4	Blank cards⁎	2	5, 6		
5	$\left\{p_{10},p_{11}\right\}$	2	7, 8	7,5	4464
6	$\left\{p_{12},p_{13}\right\}$	2	9, 10	9,5	5655
7	$\left\{p_{7}\right\}$	1	11	11	6548
8	$\left\{p_{16},p_{17}\right\}$	2	12, 13	12,5	7441
9	$\left\{p_{9}\right\}$	1	14	14	8333
10	Blank cards⁎	2	15, 16		
11	$\left\{p_{14}\right\}$	1	17	17	10,119
12	$\left\{p_{2}\right\}$	1	18	18	10,714
13	$\left\{p_{6},p_{8}\right\}$	2	19, 20	19,5	11,607

⁎ adding blank cards is the decision-maker dependent; it is the part of the Simos method; it strengthen the influence of the properties of higher importance.

Normalized weights $w_{i}$ (that can be used in SAW method) are calculated according to the formula (11).

(11) $$w_{i}=\frac{nn_{i}}{\sum_{i=1}^{n}nn_{i}}$$

where $nn_{i}$ is non-normalized weight of criterion i.

The weights’ calculation in AHP starts with mutual pairwise comparisons of every two criteria. Having them ranked with the proposed MDSM and presented in Tables 2 and 3, the level of importance can be added as a separate column (see Table 4)

**Table 4:** Levels of importance.

Ordered Criteria	Level of importance (high to low)
$\left\{p_{6},p_{8}\right\}$	1
$\left\{p_{2}\right\}$	2
$\left\{p_{14}\right\}$	3
$\left\{p_{9}\right\}$	4
$\left\{p_{16},p_{17}\right\}$	5
$\left\{p_{7}\right\}$	6
$\left\{p_{12},p_{13}\right\}$	7
$\left\{p_{10},p_{11}\right\}$	8
$\left\{p_{1}\right\}$	9
$\left\{p_{5}\right\}$	10
$\left\{p_{3},p_{4}\right\}$	11

If a pair of properties lays on the same level (like $p_{6},p_{8}$), their assessment will be 1:1. The maximum absolute value of a difference of two different levels is 10. Let's assign the figures allowed by AHP to the absolute value of a difference of two different levels (see Table 5).

**Table 5:** Assignment of AHP figures.

Absolute value of a difference of two different levels	AHP figure
1	3
2	3
3	3
4	5
5	5
6	5
7	7
8	7
9	9
10	9

Then to assess the ratio for any pair of properties (other than lying on the same level) the following procedure can be applied:

- from two comparing properties take a property of lower level of importance (see Table 4),
- assign to it value 1,
- based on Table 4 calculate absolute value of a difference of levels of importance (of the pair of properties),
- based on this absolute value and Table 5, assign the figure to the property (being compared) of higher importance level.

Applying that, the pair $p_{2}:p_{1}$ can be assessed as 7:1. The importance level of the property $p_{1}$ is lower than $p_{2}$, so $p_{1}$ is assessed as 1. The absolute value of difference of the levels is 9–2 = 7. As to the difference of 7 (left column in Table 5), the figure 7 is assigned (right column), $p_{2}$ is assessed as 7. Similarly $p_{1}:p_{2}$ will get 7:1 ratio. Other examples of the assessment: $p_{14}:p_{9}=3:1$; $p_{5}:p_{9}=1:5$; $p_{8}:p_{5}=9:1$.

This procedure – based on ordering with the use of the proposed MDSM – protects from inconsistent assignments in AHP (the example is presented in the previous section). As the negative results are not published the authors couldn't published their earlier research, where only 7 criteria were mutually assessed by 163 respondents. Only 4 sets of answers were consistent enough to continue the calculation of weights with AHP method.

## Ethics statements

Not applicable

## Funding

This work was supported by Studio Vector S.A. executing the grant “A multi-module system for optimizing the epidemic safety management of public buildings” number POIR.01.01.01–00–2440/20; the National Centre for Research and Development, Warsaw, Poland.

## CRediT authorship contribution statement

**Hubert Anysz:** Conceptualization, Methodology, Visualization, Writing – original draft, Validation. **Bartosz Grucza:** Conceptualization, Methodology, Writing – review & editing, Supervision.

## Declaration of Competing Interest

The authors declare that they have no known competing financial interests or personal relationships that could have appeared to influence the work reported in this paper.

## Data Availability

No data was used for the research described in the article.
